# Impact of first metatarsal shortening on forefoot loading pattern: a finite element model study

**DOI:** 10.1186/s12891-019-2973-6

**Published:** 2019-12-27

**Authors:** Xiang Geng, Jiaqi Shi, Wenming Chen, Xin Ma, Xu Wang, Chao Zhang, Li Chen

**Affiliations:** 1 Department of Orthopedics, Huashan Hospital, Fudan University, 12 Middle Wulumuqi Rd., Jing’an District, Shanghai, China; 2 Academy for Engineering & Technology, Fudan University, Shanghai, China; 3 School of Medical Instrument and Food Engineering, University of Shanghai for Science and Technology, Shanghai, China

**Keywords:** First metatarsal shortening, Plantar pressure, Finite element method, Hallux valgus, Transfer metatarsalgia

## Abstract

**Backgrounds:**

There has long been a consensus that shortening of the first metatarsal during hallux valgus reconstruction could lead to postoperative transfer metatarsalgia. However, appropriate shortening is sometimes beneficial for correcting severe deformities or relieving stiff joints. This study is to investigate, from the biomechanical perspective, whether and how much shortening of the first metatarsal could be allowed.

**Methods:**

A finite element model of the human foot simulating the push-off phase of the gait was established. Progressive shortening of the first metatarsal from 2 to 8 mm at an increment of 2 mm were sequentially applied to the model, and the corresponding changes in forefoot loading pattern during push-off phase, especially the loading ratio at the central rays, was calculated. The effect of depressing the first metatarsal head was also investigated.

**Results:**

With increasing shortening level of the first metatarsal, the plantar pressure of the first ray decreased, while that of the lateral rays continued to rise. When the shortening reaches 6 mm, the load ratio of the central rays exceeds a critical threshold of 55%, which was considered risky; but it could still be manipulated to normal if the distal end of the first metatarsal displaced to the plantar side by 3 mm.

**Conclusions:**

During the first metatarsal osteotomy, a maximum of 6 mm shortening length is considered to be within the safe range. Whenever a higher level of shortening is necessary, pushing down the distal metatarsal segment could be a compensatory procedure to maintain normal plantar force distributions.

## Background

Transfer metatarsalgia is one of the most common complications of the hallux valgus reconstruction procedure, which often lowers patients’ satisfaction rate [1]. For a long time, shortening of the first ray has been considered to be the main cause of pain due to transfer metatarsalgia [2]. Thus, most surgeons [3–5] tend not to shorten the first metatarsal, regardless of the procedures they choose.

Maestro et al. [6] proposed the concept of harmony metatarsal, which advocated the first metatarsal should be at a similar length with the second one, or shorter within 2 mm. However, a dissenting view began to emerge in recent years. Ahn et al. [7] found no significant correlation between the shortening and postoperative transfer metatarsalgia. Moreover, appropriate shortening is indeed required including in patients with severe deformities or those suffering from stiff joints. So the debate has increased gradually about whether and how much the first metatarsal shortening could be allowed during hallux valgus correction.

More recently, Geng et al. [8] found that abnormal loading patterns on the forefoot were the direct reason for transfer metatarsalgia. Using the plantar pressure data, they defined a loading ratio of the central rays to the whole forefoot: The foot was considered to be at high risk for transfer metatarsalgia whenever this ratio exceeds 55% during the push-off phase. However, other factors apart from the metatarsal length may also alter the forefoot biomechanics including elevation of the first metatarsal, subluxation of sesamoids, and hypermobility of the first metatarsal-cuneiform joint.

Nevertheless, in vivo human studies are nearly impossible to get rid of the confounding factors mentioned above. When evaluating the effects of length changes on clinical outcomes, none of those studies focused on the intrinsic biomechanics changes caused by shortening of the first metatarsal [3–5, 7]. Therefore, different studies tend to draw different conclusions, and we believe that this controversy may endure if studies merely focus on changes in length. Thus, we performed 3D finite element analysis and investigated the relationship between shortening and corresponding plantar loading patterns to determine whether and how much shortening of the first metatarsal could be allowed to avoid transfer metatarsalgia.

## Methods

### Finite element model

The study was approved by the institutional review board of our hospital. A volunteer was recruited, who had been informed of the purpose, methods and potential risks of our study and gave consent to participate. The subject’s weight-bearing dorsal-plantar radiography showed no foot deformities. Both of his first metatarsals were the same relative length. To obtain an FE model simulating the push-off phase of the gait, the dorsiflexion degrees of ankle and metatarsophalangeal (MTP) joint were acquired precedently. The subject’s foot kinematics were measured using the multi-segment Oxford Foot Model (Vicon Motion Systems (Oxford Metrics, Oxford, UK)). When the right foot reached its peak loading at the central rays during push-off, the dorsiflexion of its ankle and MTP joint averaged 9 degrees and 19 degrees, respectively.

**Fig. 1:**
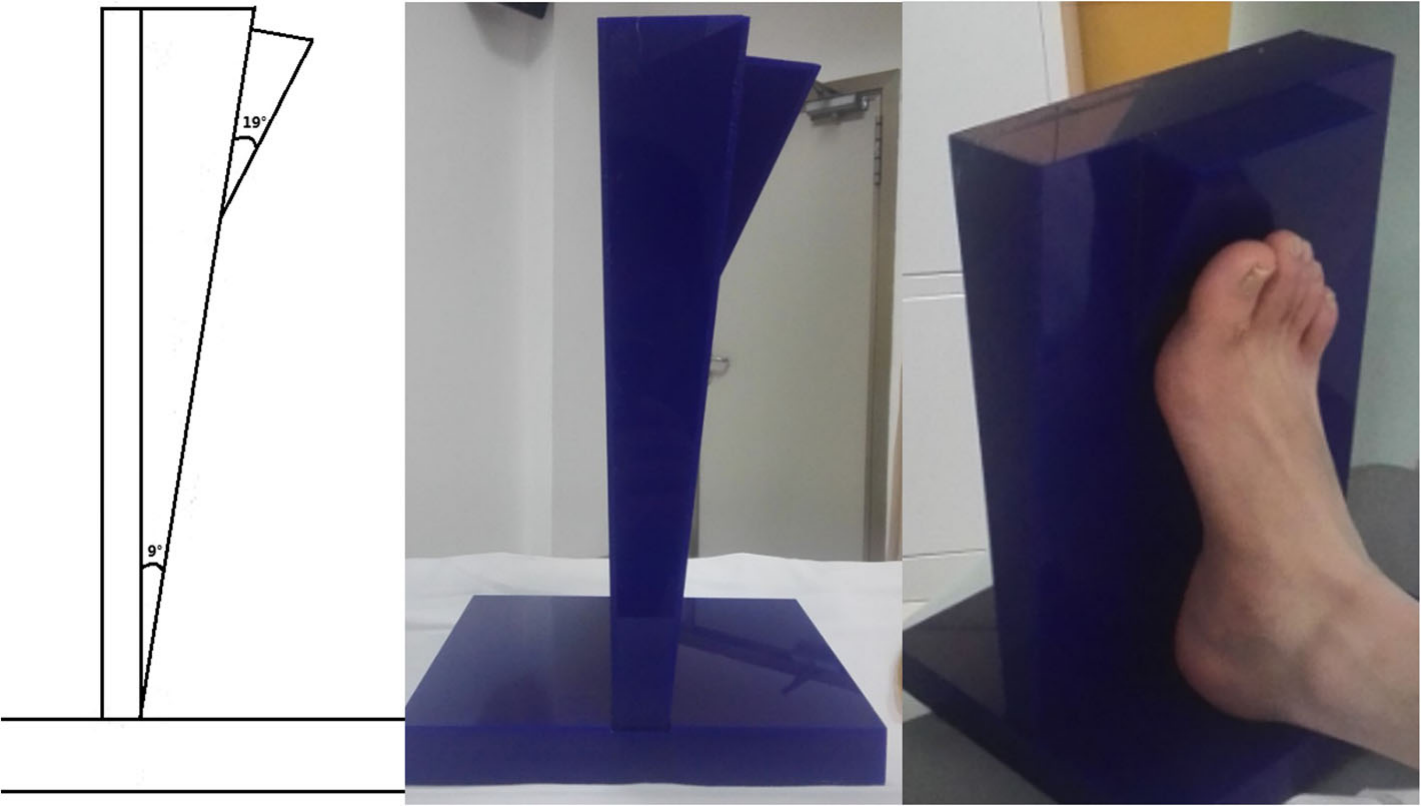
Customized foot plate used to maintain the joints in position during CT scans. As shown in the figure, the dorsiflexion of its ankle and MTP joint was controlled at 9 degrees and 19 degrees respectively

Next, non-weight bearing CT scans were performed on the subject’s right foot with a customized foot plate that was designed to maintain the ankle and MTP joints in designated positions. This mimics the push-off phase in the gait to acquire a bony structure matching the real push-off phase (Fig. 1).

The CT images were obtained contiguously at 0.67-mm resolution (100 kV × 80 mA, volume EC, 512 × 512 matrix). Images were then segmented in the software, Mimics 17.01 (Materialise, Leuven, Belgium). Three-dimensional solid parts were constructed for 30 bones including two sesamoids under the first metatarsal, and the encapsulated bulk soft tissue. Two-dimensional shell surfaces were constructed on ligaments based on their anatomic site and confirmed with orthopedic surgeons.

The bone tissue was assumed to be isotropic and homogeneous. It was assigned to an elastic modulus of 7300 MPa with a Poisson’s ratio of 0.3 [9]. The bony joints were passively stabilized by 134 major ligaments and a fan-like plantar fascia. The material properties of the ligaments and plantar fascia was defined according to previous studies [10, 11]. The cross-section area of the ligaments was assigned with 18.4 mm^2^ as reported by Mondal et al. [10].

**Fig. 2:**
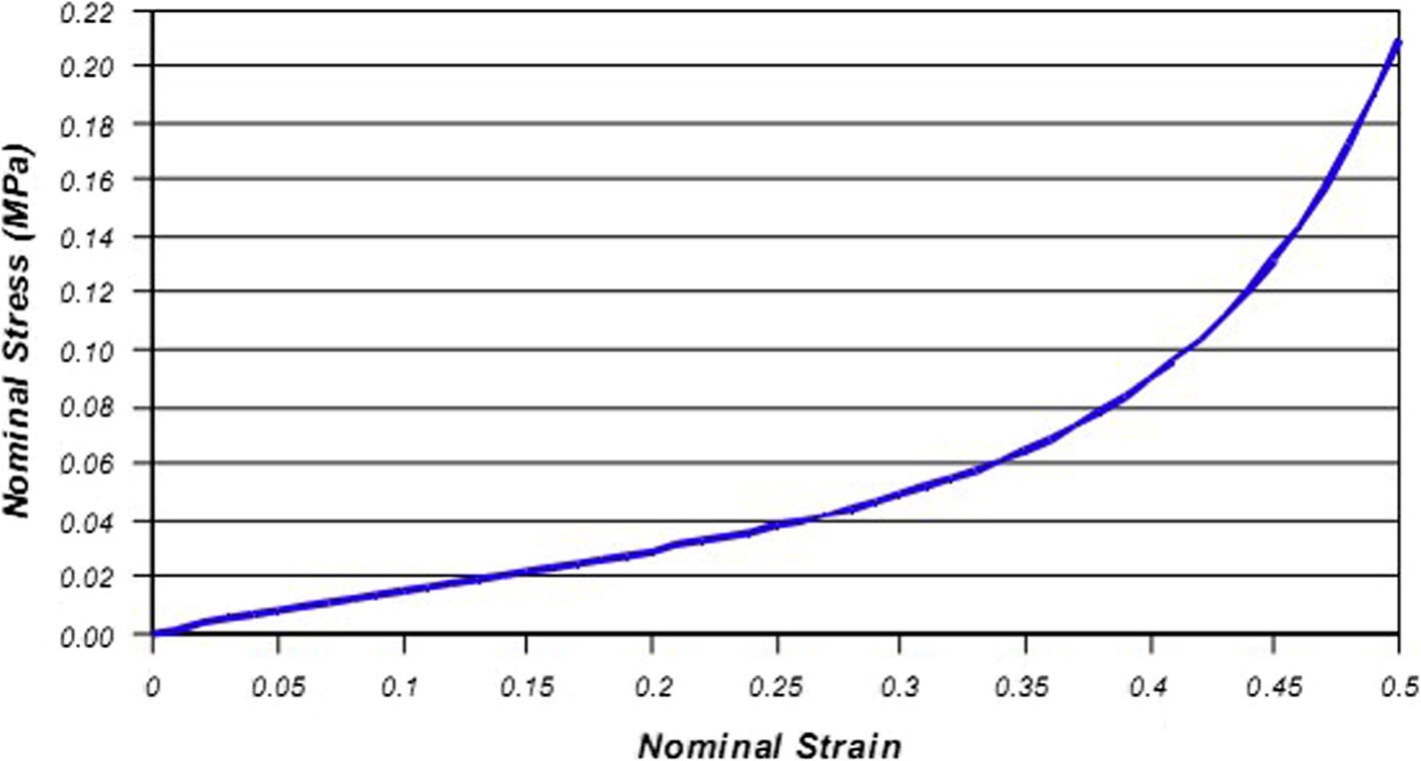
Stress–strain behavior of plantar soft tissue represented by the ogden hyperelastic model

The supporting ground included two layers. The bottom layer was defined as rigid, and the upper layer was assigned to 40 GPa representing concrete ground [12]. According to Chen et al. [13, 14], plantar soft tissue was modeled with an incompressible Ogden hyperlastic material (Fig. 2)— a constitutive model that has previously been used to model nonlinear plantar soft tissue material behavior. The material’s parameters are given in Table 1.

**Table 1:** Material properties used for the different tissues

	Elastic modulus (Mpa)	Poisson ratio
Bone [9]	7300	0.3
Ligament [10]	268.4	0.4
Plantar fascia [11]	350	0.4
Plantar soft tissue [13, 14]	1st-order Ogden hyperlastice model (μ _1_ = 0.0375 Mpa and α _1_ = 5.5)	

**Fig. 3:**
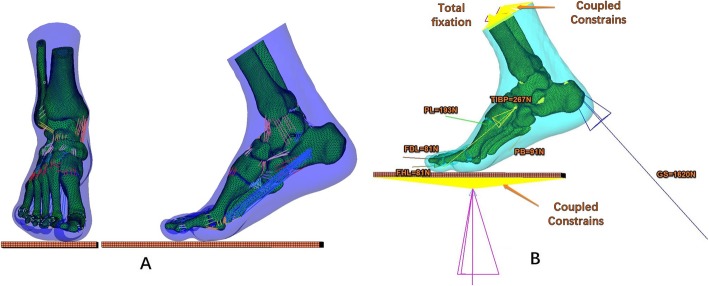
Completed finite element model. **a**: AP and lateral view of the finite element model of the foot, mimicking a push-off pose in gait. **b**: Simulated load and boundary conditions

The entire foot model including the bony parts and plantar soft tissues were meshed in ABAQUS. The total number of elements is 354,325 (108,262 nodes); the element size was optimized based on mesh convergence analysis. (Fig. 3).

To simulate the muscular loads, six major flexor muscles are included: the gastrocnemius–soleus (GS) complex, tibialis posterior (TIBP), flexor halluces longus (FHL), flexor digitorum longus (FDL), peroneus brevis (PB), and peroneus longus (PL). Muscles were modeled as one-dimensional connectors. Muscle selection and their muscle forces were adopted from Chen et al. [14], including GS = 1620 N, TIBP = 267 N, FHL = 130 N, FDL = 81 N, PB = 91 N, and PL = 193 N. (Fig. 3) The ground reaction force (939.6 N) at the moment when the central rays reached their maximum loading was applied through the ground; meanwhile the tibia and fibula were constrained to make coupled movements. Frictionless contact was assumed in all internal foot joint. The coefficient of friction in the ground contact was 0.6. These loading protocols were solved using ABAQUS (SIMULIA), and the plantar force distribution was calculated.

For model validation, plantar pressure data of the same subject using plantar pressure measuring system (Footscan, Rscan, Belgium) was obtained. To precisely compare between the experimental and numerical results, we divided the forefoot plantar area into 5 masks including the hallux (T1), the second to fifth toe (T2–5), metatarsals 1 (M1), metatarsals 2 to 3 (M2 + 3), and metatarsals 4 to 5 (M4 + 5). We then compared the FE calculated results against the volunteer’s actual plantar pressure data to validate the FE model.

### Simulated first metatarsal shortening procedure

**Fig. 4:**
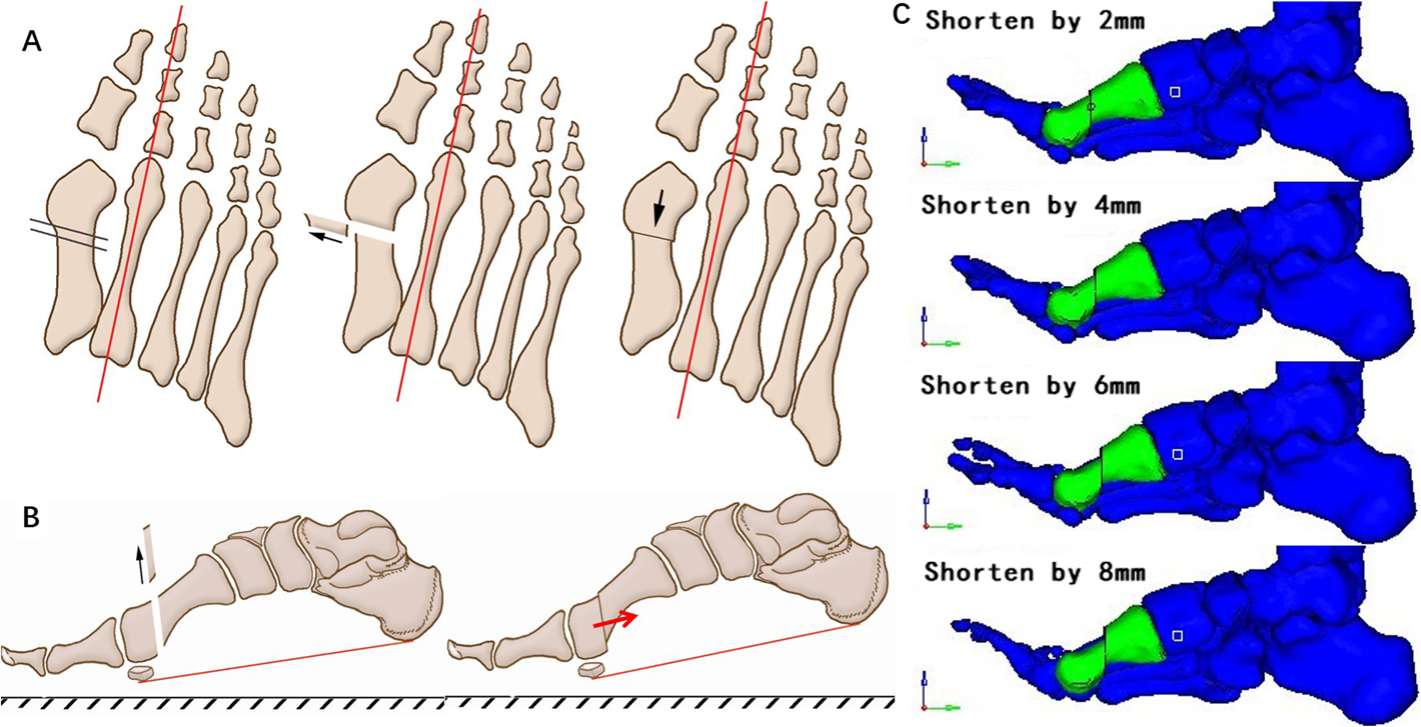
Simulated osteotomy procedure for the first metatarsal. **a**: AP view of osteotomy and movement of the bone segments. The osteotomy was perpendicular to the longitudinal axis of the second metatarsal, while the movement direction was parallel to the axis. **b**: A lateral view of osteotomy and movement. The osteotomy was perpendicular to the reference plane, while the movement direction was parallel to the plane. **c**: Lateral views of metatarsal bones shortened by different length in FE mode

Shortening length is the only variable discussed in this study, and thus we performed a standardized metatarsal shortening procedure based on the following protocol to eliminate the confounding factors such as differences in osteotomy methods or intrinsic structural deformities seen in pathological foot conditions. First, the lowest points of the calcaneus—the sesamoid under the first metatarsal and the fifth metatarsal—were chosen as the reference points to determine the horizontal reference plane. Second, osteotomy of the first metatarsal was made perpendicular both to the longitudinal axis of the second metatarsal and to the reference plane. The shortening length was determined to be 2 to 8 mm according to former studies on postoperative metatarsal length at an increment of 2 mm. After the osteotomy procedure, the distal part of the metatarsal segment was moved proximally and parallel to both the longitudinal axis of the second metatarsal and the reference plane mentioned above. Two parts of the metatarsal were then fixed to be a new shortened part (Fig. 4).

**Fig. 5:**
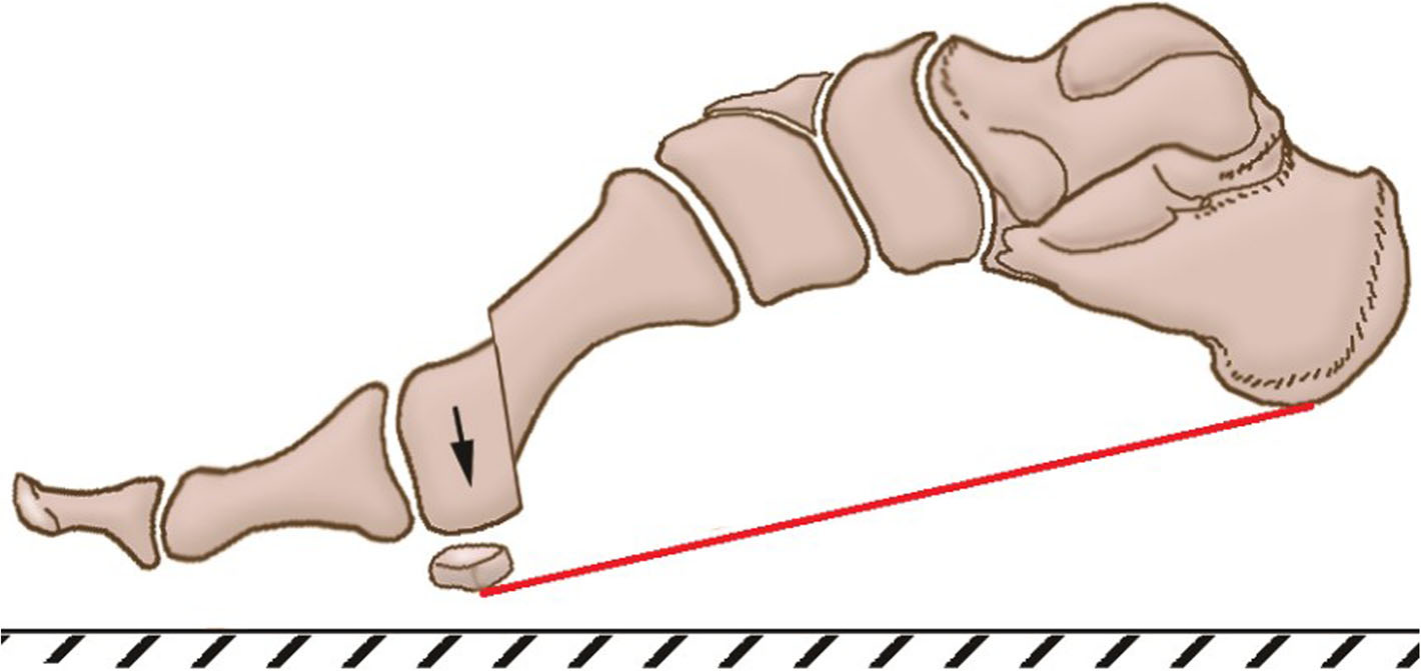
Pattern for distal end pushing down. The pushing down direction is perpendicular to the reference plane

Surgical treatment for severe joint malformation or osteoarthritis often leads to a substantial length change of the first metatarsal. In these scenarios, the osteotomy procedure known as “pushing down motion” for the distal end of the metatarsal might be a useful approach to prevent transfer metatarsalgia. In order to validate its effectiveness, we also simulated the “pushing down motion” following the osteotomy procedure. In addition, the risk of metatarsalgia raises substantially when the central ray plantar loading ratio reaches 55% [8]. Thus, when first metatarsal shortening reaches a certain extent (the central ray plantar loading ratio exceeds 55%), the distal end of the first metatarsal will be compensatively pushed down by 3 mm before being surgically fixed to the proximal part. The pushing down direction is perpendicular to the reference plane. (Fig. 5) The same loading protocol mentioned above was applied to all models with different osteotomy procedures, and the plantar pressure distribution was calculated for individual models.

## Results

### FE model validation

**Fig. 6:**
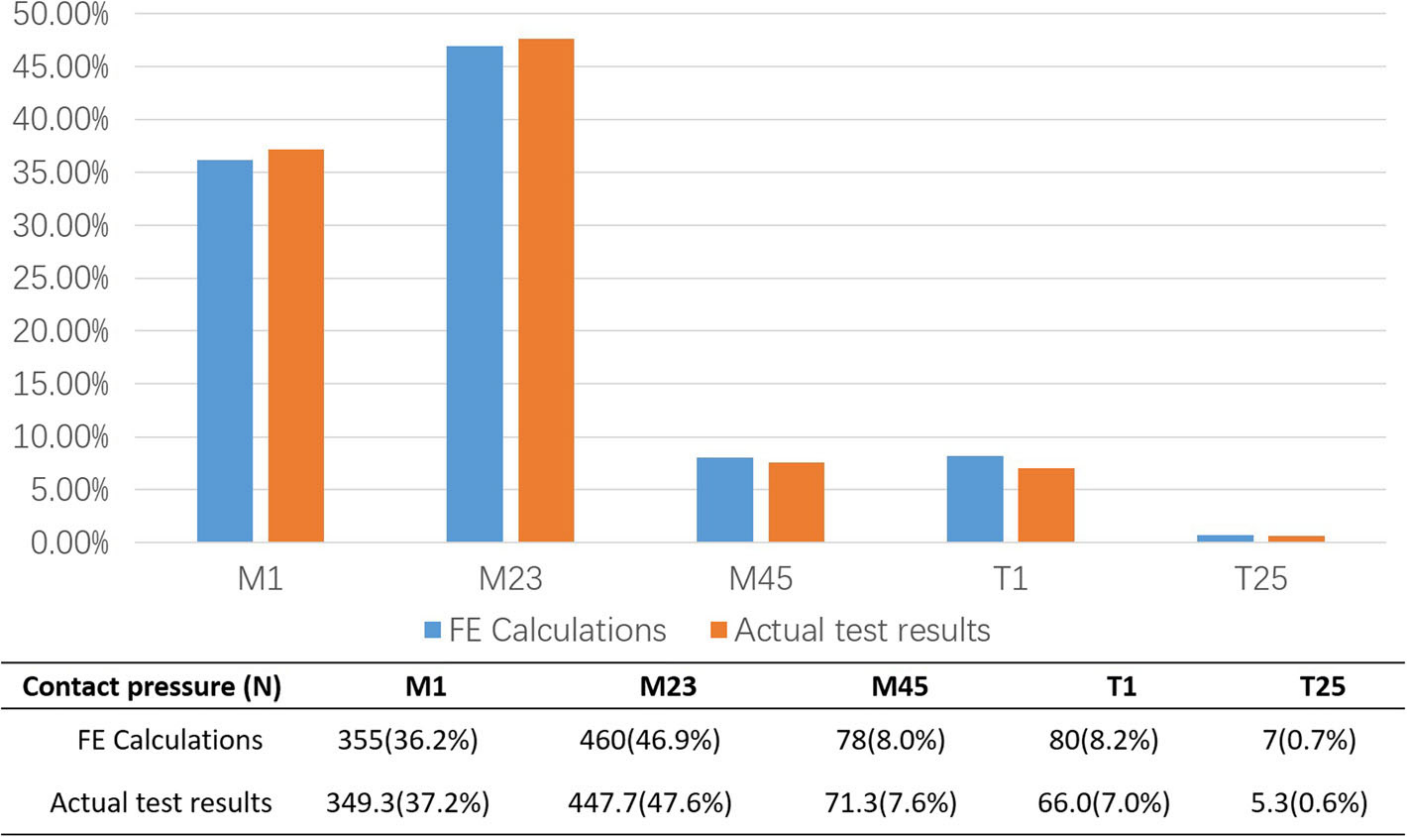
Intact FE model results compared against subject’s actual plantar pressure distribution

The calculated results of the FE model and the volunteer’s actual plantar pressure showed reasonable agreement (Fig. 6). The model-calculated results matched the actual testing data in terms of the peak plantar pressure values. This indicates that the developed foot FE model is effective in calculating the peak plantar pressure as well as the pressure distribution.

### Model sensitivity analysis

**Fig. 7:**
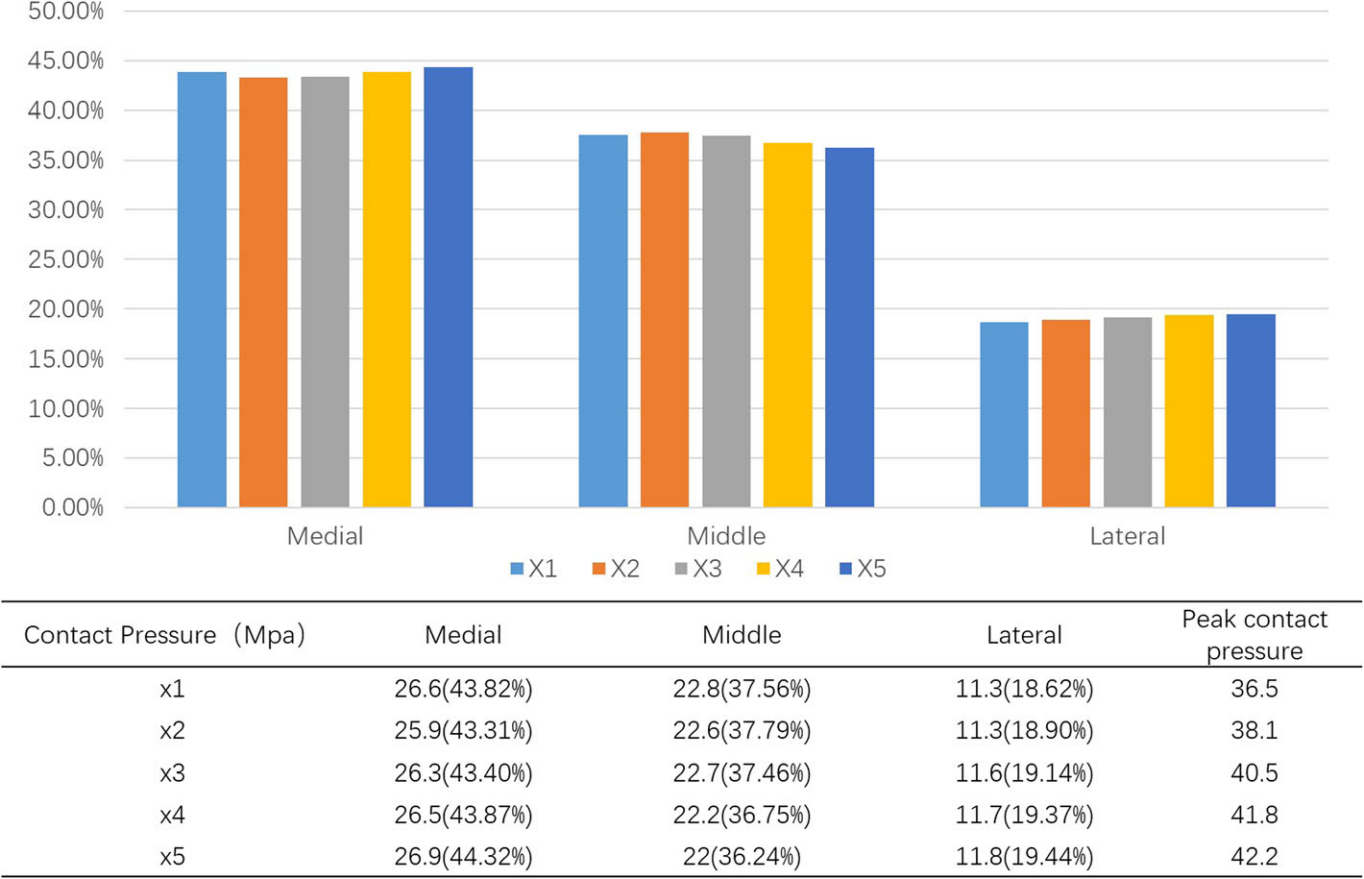
FE model sensitivity test. While material hardness increases to five times the original, plantar pressure proportion of each part of forefoot all show great stability, without substantial tendency of increase or decrease

The elastic property of the plantar fat pad is subject to changes in pathological foot conditions. According to a recent study [15], patients’ elastic modulus of forefoot plantar soft tissue increases 4-times the normal value on average. To account for the potential impact of material characteristics on model results, we conducted a sensitivity test by altering the material properties of the plantar soft tissue up to 5 times that of the original hardness [16]. The results (Fig. 7) showed that despite the different material properties, the plantar pressure distribution is consistent with no substantial changing tendency—this proves the sensitivity of our FE model.

### Impacts of metatarsal shortening on plantar pressure

**Fig. 8:**
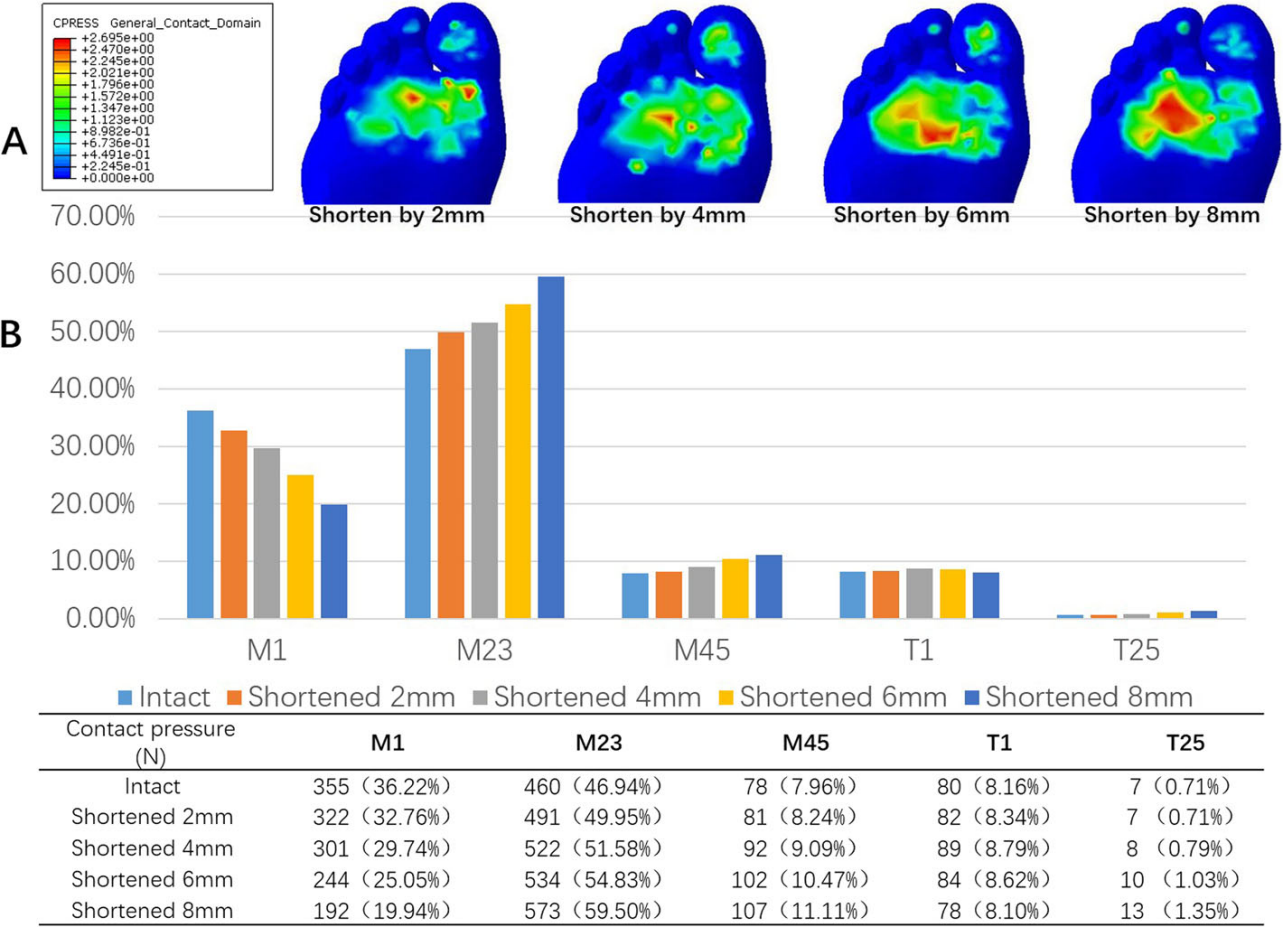
Impact of first metatarasal bone shortening on forefoot loading. **a**: Changes in forefoot plantar pressure distribution in response to first metatarsal shortening. As is shown in the figure, with the shortening level increasing, the pressure of the lateral rays continued to rise **b**: Comparison of contact pressure of each area with intact metatarsal bone and with metatarsal shortened by different lengths

During the first metatarsal shortening, the first ray plantar pressure gradually decreased, while the second and third ray, the fourth and fifth ray, and the toe area plantar pressure increased. With increased shortening level, the plantar pressure of the first ray decreased rapidly, while the pressure of the other rays continued to increase. Referring to the loading ratio, the second and third rays—also known as central rays—accounted for 49.9, 51.6, 54.8, and 59.5% of the total weight of the forefoot when shortened to 2 mm, 4 mm, 6 mm, and 8 mm, respectively. This shows a considerable increasing trend (Fig. 8). These results showed that only when the shortening reaches 6 mm or more does the loading ratio of the central rays during push-off exceed 55%.

### Impacts of distal segment “pushing down motion” on plantar pressure

**Fig. 9:**
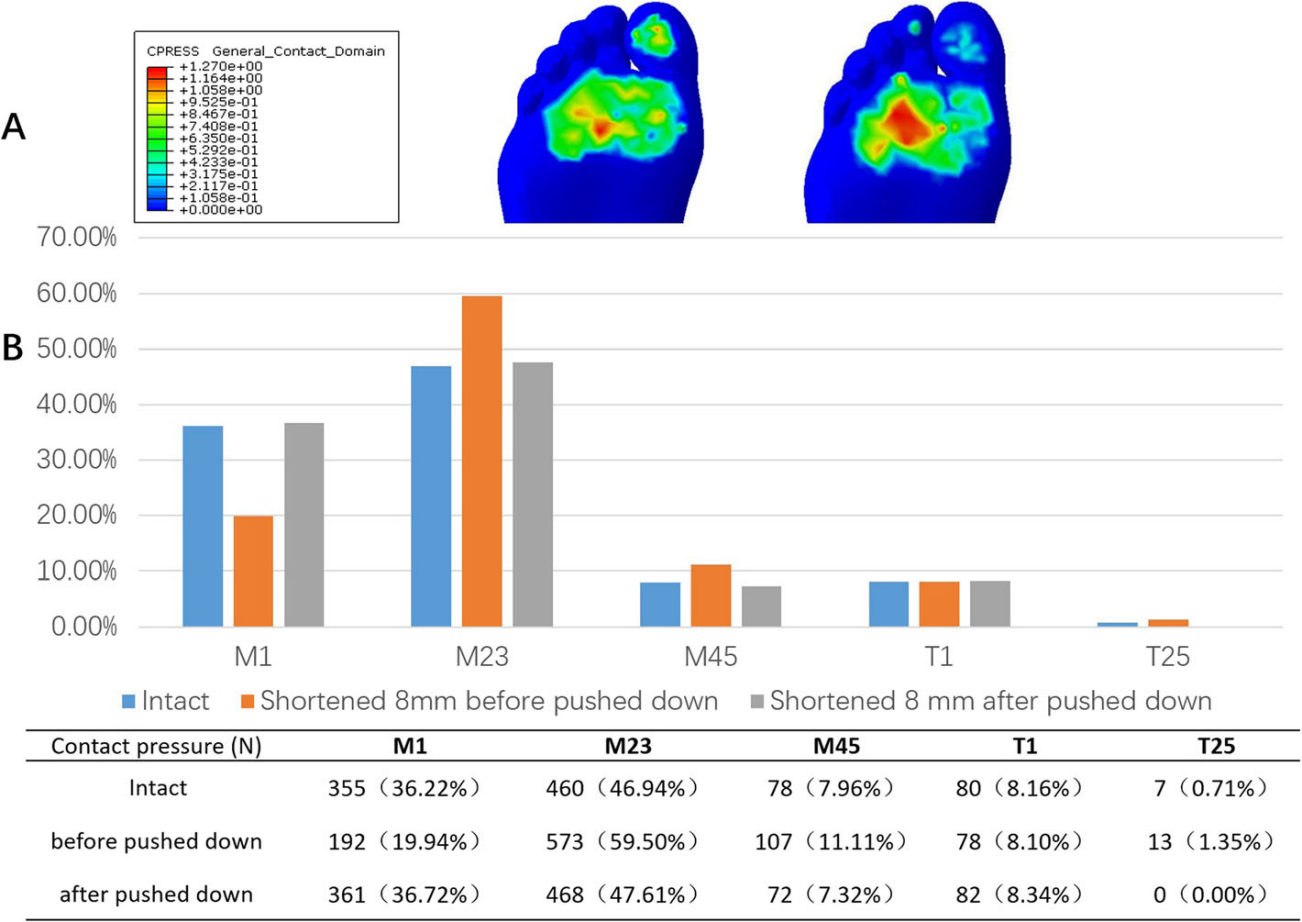
Impact of “pushing down motion” on forefoot loading. **a**: Changes in forefoot plantar pressure distribution in response to distal segment being pushed down by 3 mm. As is shown, after distal segment “pushing down” motion, the pressure of the lateral rays decreased. **b**: Comparison of contact pressure of each area with or without compensative distal end pushing down

To demonstrate the compensative function of depressing the head of the first metatarsal, we pushed down the distal segment and observed changes in plantar pressure distribution. The loading ratio decreased from 59.5 to 47.6% after an 8-mm shortening when the distal segment of the first metatarsal was lowered by 3 mm. This is very close to the normal situation (Fig. 9).

## Discussion

The basic biomechanical abnormality for transfer metatarsalgia is manifested as an alteration of forefoot loading pattern. A previous study by Geng et al. [8] on post-osteotomy patients showed that the risk of metatarsalgia increased substantially when the plantar loading ratio of the central rays reached 55% in the push-off phase during gait. Therefore, this study focuses on the effect of magnitudes of the first metatarsal shortening on the plantar pressure distribution during push-off. Moreover, by using the forefoot loading ratio of 55% as a threshold value, we might determine the optimal value for the first metatarsal shortening to reduce risks.

Standardized FE simulations for metatarsal shortening procedure were performed in a healthy foot rather than a diseased one. In this situation, we assume all the alignment and other structural deformities have been corrected, so the confounding factors due to different deformities that may potentially influence model results are excluded. Furthermore, our study is not intended to evaluate the efficacies of certain osteotomy procedures for hallux valgus correction. Rather, we carefully designed a method that is not applied during clinical routine, but can purely simulate the effects of the first metatarsal shortening. All of these ensure this study to focus on potential relationships between the level of metatarsal shortening and alterations in plantar pressure patterns.

The three-dimensional finite element model established here contains relatively complete tissue structures. The soft tissue is set as a hyperelastic material ensuring the simulation and structural completeness of the model. In addition, the model reflects an actual forefoot push-off phase, and both the dorsiflexion angles of the ankle and the first metatarsophalangeal joint during push-off are reproduced. Validation with plantar pressure results is promising.

Our results showed that the weight-bearing ratio of the first ray was gradually decreased with the increased shortening length when the first metatarsal is shortened; the central rays and the lateral rays’ plantar pressure increased. We speculate that the contact area of the first ray during push-off decreases when the first metatarsal is shortened. This leads to more weight transfer to the central rays and lateral rays. These results meet our expectations.

The loading ratio of the central rays increased up to 54.8% when the first metatarsal was shortened by 6 mm. This is close to the 55% that is thought to be a risky threshold based on previous studies [8]. In other words, when the first metatarsal shortening is less than 6 mm, the increased loading ratio of the central rays does not reach the critical value for transfer metatarsalgia. To avoid simultaneous elevation of the first metatarsal head, the shortening direction is parallel to the sole of the foot. In addition, since the first metatarsal of the model volunteer is at relatively the same length as the second, we can conclude that the first metatarsal should be controlled to be 6 mm shorter than the second metatarsal.

In this study, the central rays loading ratio reached 59.5% when the shortening reached 8 mm. This resulted in a high risky foot with postoperative transfer metatarsalgia. However, we noticed that if the distal end of the first metatarsal is depressed further (“push down motion”) by 3 mm, then its plantar pressure could be restored considerably, i.e., the central rays’ loading ratio again dropping to 47.6%. This suggests that if a larger magnitude of shortening of the first metatarsal is necessary during surgery, then we shall compensate for its loss of weight-bearing function by appropriately depressing its distal bone segment.

Carr and Boyd [17] argued that the degree of the first metatarsal shortening in the treatment of hallux valgus should not exceed 4 mm. In this study however, the weight-bearing ratio of the central rays only increased to 51.6% when the first metatarsal was shortened by 4 mm. This gap may be associated with the shortening method in their study—it simultaneously elevates the distal end while also leading to shortening—both of these impair the weight-bearing function of the first ray during the push-off phase. However, the shortening done here is parallel to the sole of the foot, and it does not involve any elevation in the sagittal plane. Thus, the safe range of shortening is relatively larger.

Zhang et al. [4] reported that the first metatarsal was shortened on average by 1.8 mm with the maximum shortening no more than 6 mm. They found that the incidence of postoperative transfer metatarsalgia increased with the degree of shortening, but they failed to describe sagittal elevation or depression of the distal end. Their measurement method connected the central points of the proximal to the distal articular surfaces of the first metatarsal; thus, they focused on absolute rather than relative length, which we believe has less clinical relevance.

Toth et al. [3] showed an average shortening of 3.8 mm of the first metatarsal in their operation, while the average depression of the distal end was 2.7 mm. They concluded that the shortening of the first metatarsal was significantly associated with postoperative subjective pain scores. However, the authors failed to record whether the patients with postoperative pain were accompanied by recurrence of hallux valgus, malunion, dislocation of metatarsophalangeal joint, or hammer toe deformity—this could also cause postoperative pain despite less shortening.

There have been previous reports of a relatively large degree of first metatarsal shortening. Klosok et al. [18] reported an average shortening of the first metatarsal by 10 mm after surgery for hallux valgus. Keogh et al. [19] followed up hallux valgus cases and found an average shortening of the first metatarsal by 5 mm. Pouliart et al. [20] reported an average shortening of 8.5 mm after the operation. However, none of these studies found any definite correlation between the degree of shortening and the incidence of transfer metatarsalgia. From a biomechanical viewpoint, our simulation provides a theoretical basis for these clinical reports because when the first metatarsal distal end height stays unchanged, the central rays’ loading ratio will not increase to a relatively risky range until the shortening exceeds 6 mm. Furthermore, even larger length of shortening might be allowed if its distal end is pushed down.

The factors affecting the distribution of forefoot weight-bearing are certainly not due only to the length of the first metatarsal. In this study, however, we try to rule out the poor alignment of first ray by modeling a normal foot including the first tarsometatarsal joint relaxation, sesamoid dislocation, and other related factors. We focused solely on the length change of the first metatarsal to provide guidelines for osteotomy surgical strategy. With more research, patient-specific models may emerge to improve this complicated procedure.

### Limitation

There are some limitations to this study. The simulation of osteotomy is rare in the literature. It is designed, however, according to the direction of the longitudinal axis of the second metatarsal and a reference plane that assures the anatomical axis of the first metatarsal remained unchanged. Also, this biomechanical study is still based on a quasi-static model that merely focuses on a certain moment during push-off. Finally, while our FE model was validated by plantar pressure data, it cannot replace in vivo studies. The inter-individual variability and other in vivo compensatory mechanisms are not considered. Therefore, quantitative results from this study only provide general guidelines for surgeons to perform first metatarsal shortening procedures during the hallux valgus reconstruction surgery. Further clinical studies on patient populations are needed to validate these conclusions, as well as to determine whether and how they can be precisely applied to different populations.

## Conclusions

In conclusion, our study shows that 6 mm of shortening of the first metatarsal is within a safe range during hallux valgus operation. If more shortening is needed, its distal end should be depressed appropriately to prevent post-operative transfer metatarsalgia.

## Data Availability

The datasets used and/or analysed during the current study are available from the corresponding author on reasonable request.

## Abbreviations

CT: Computer Tomography
DCMO: Distal Chevron metatarsal osteotomy
FE: Finite Element
MTP: Metatarsophalangeal
PCMO: Proximal Chevron metatarsal osteotomy
